# COVID-19 and diabetes in 2020: a systematic review

**DOI:** 10.1186/s40545-023-00546-z

**Published:** 2023-03-09

**Authors:** Farideh A. Javid, Fadi Abdul Waheed, Nisa Zainab, Hamza Khan, Ibrahim Amin, Ammar Bham, Mohammed Ghoghawala, Aneem Sheraz, Radi Haloub

**Affiliations:** 1 Department of Pharmacy, University of Huddersfield, Huddersfield, HD1 3DH UK; 2 Biotech and Pharmaceutical Management, Global Business School for Health (GBSH), University College London (UCL), London, UK

**Keywords:** COVID-19, Diabetes, Ethnicity, Risk factors

## Abstract

Attempts were made to review the literature on diabetic patients who experience complications when they contract COVID-19, and to determine whether ethnicity and other risk factors play an important role in the development of symptoms and their severity, as well as responding to medications. A literature search was performed using five keywords, namely COVID-19, diabetes, ethnicity, medications, and risk factors between January 2019 and December 2020 using electronic databases such as PubMed, Science Direct, Google Scholar, Springer Link, and Scopus. Forty studies were included. The review indicated that diabetes was a significant risk factor for poorer outcomes and increased mortality associated with COVID-19. There were several risk factors for diabetic patients that increased their likelihood of poorer outcomes associated with COVID-19. These included black and Asian ethnicity, male sex with high BMI. In conclusion, patients with diabetes of black or Asian origin with high BMI, male sex, and older age had an increased risk of poorer outcomes associated with COVID-19. This highlights the importance of considering the history of the patient in prioritising care and treatment.

## Introduction

In 2019 a new strain of coronavirus was released known as ‘COVID-19’ from Wuhan, China. This new strain of coronavirus, named SARS-COV-2, rapidly spread and the WHO announced a worldwide pandemic within a matter of months. There has been a total of 58,274,308 reported cases and 1,382,745 deaths worldwide and in the United Kingdom a total of 1,512,045 reported cases and 55,024 deaths from COVID-19 up to December 2020 [1]. The virus has shown to be particularly deadly as it has a high rate of transmission. Evidence has shown that the most common symptoms include fever, dry cough, headache, fatigue, shortness of breath, sore throat, muscle aches, as well as gastrointestinal (GI), cardiovascular system (CVS), and central nervous system (CNS) disturbances. More severe symptoms are seen in the elderly and patients with comorbidities. These patients commonly experience severe complications, which can ultimately lead to death. While some patients are asymptomatic, they can be a carrier of the virus, which further complicates the control of transmission, particularly in individuals with comorbidities such as patients diagnosed with diabetes. Some studies have shown a higher mortality rate in diabetic patients when compared to non-diabetic patients. The increase in the mortality rate has led us to review the variables that contributed to the high mortality rate of COVID-19 in diabetic patients that were published up to the date of December 2020. Furthermore, this article suggests the appropriate healthcare approach after the early diagnosis of COVID-19 for diabetic patients and the interventions that the healthcare sector can consider reducing the risk of mortality.

## Method

The Preferred Reporting Items for Systemic Reviews and Meta-Analysis (PRISMA) guidelines were followed. Briefly, a literature search was performed between January 2019 and December 2020. The following databases were searched: PubMed, Science Direct, Google Scholar, Springer Link, and Scopus.

### Search strategy

**Fig. 1:**
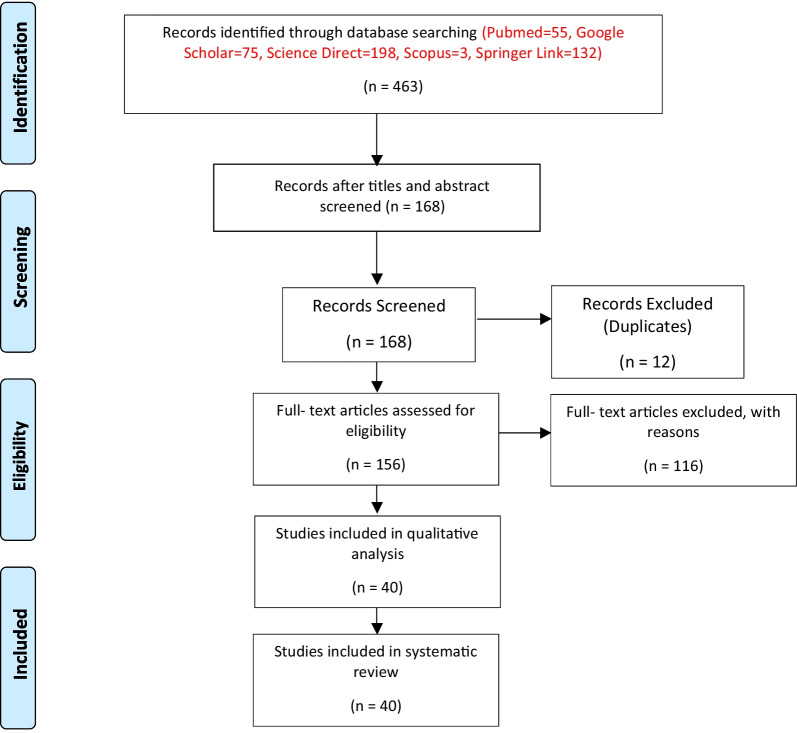
PRISMA flow diagram showing the process of screening, selection and inclusion of relevant articles to the study

The following five keywords were included in the search in all databases: “COVID-19” AND “Diabetes” AND “Ethnicity” AND “Risk factors”. Duplicates were removed using ‘OR’ as appropriate and where possible. Original research articles were included. The exclusion criteria included papers in other languages than English, commentaries, reviews, news reports, and conference abstracts. The process of screening, identification and inclusion for this review is shown in PRISMA chart in Fig. 1. From the search in the databases, 40 articles were within the defined criteria, that is, original research articles that were taken for further evaluation (Fig. 1).

### Inclusion and exclusion criteria

The reviewers independently screened the titles and abstracts, and selected the studies that were within the inclusion and exclusion criteria. Studies were only included if they were original research articles, available as full text, and published between January 2019 to December 2020 in English language. Anything else such as reviews, commentaries, letters to editors or abstracts presented at scientific conferences were excluded.

### Quality assessment

To avoid bias in the study, the inclusion criteria were strictly followed during the search process to provide reliable data. In addition, discussions were conducted to resolve any conflict between the reviewers and consensus was developed.

### Data extraction

The collected data including authors, year, objectives, key findings, country from eligible articles were included in Table 1.

**Table 1:** Characteristics of the relevant studies used

Number	Author(s)	Year published	Country	Study design	Aims/objectives of study	Key findings
1	Hippisley-Cox et al.	2020	England	Original Article	This study aimed at seeing whether patients who were prescribed ACEIs and ARBs would be more at risk of contracting COVID-19 severely and whether they would need to be hospitalised in the ICU	This study showed that some patients who have diabetes also take ACEIs or ARBs. But, with these medications, it showed no negative impact on COVID-19 and even led to results indicating a reduced risk of COVID-19. It also showed a link between how patients of differing ethnic minorities had differing effects to COVID-19 disease whilst being on ACEIs/ARBs
2	Sattar et al.	2020	United Kingdom	Original Article	This paper wanted to express if there was a link between BMI (High BMI risk factor of diabetes) and possible risk of COVID-19 related deaths	This study found that patients who were non-white and had a higher BMI tended to be more strongly linked with COVID-19 deaths
3	Rezende et al.	2020	Brazil	Original Article	This study aimed to identify people from the population who would be at a great risk of having severe COVID-19	This study showed that people who had diabetes, and other risk factors (hypertension, age, etc.) were more likely to have severe COVID-19
4	Zakeri et al.	2020	England (London)	Original Article	This study wanted to examine whether ethnic profile leads to an increase in severe COVID-19 or in-hospital mortality	The key findings are that Black and mixed ethnicities are at greater risk of contracting severe COVID-19, but it does not increase in-hospital stay. Socioeconomic factors may be why there is a greater risk to patients of ethnic backgrounds
5	Ho et al.	2020	United Kingdom	Original Article	This study aimed to show the different factors in which it can be explained that ethnic minorities have worse prognosis to severe COVID-19	The key finding that can be suggested from this is that lifestyle can affect prognosis of COVID-19 as well as other clinical factors. A maintenance of good lifestyle is shown to reduce the risk of contracting a severe form of the virus
6	Selden and Berdahl et al.	2020	USA	Original Article	This study aimed to show the possible link between racial/ethnic disparities seen in COVID-19	It was found that black people who lived in households with keyworkers were 1.6 times more likely to contract severe COVID-19 and with Hispanics, it showed that there was a higher percentage than black people or white people to have a severe form of the virus with someone in the household who cannot work from home
7	McGurnaghan et al.	2020	United Kingdom (Scotland)	Original Article	To ascertain the cumulative risk of fatal or critical care unit-treated COVID-19 in people with diabetes, compared with people without diabetes, and to investigate risk factors for fatal or critical care unit-treated COVID-19 among diabetic individuals	Overall risks of fatal or critical care unit-treated COVID-19 were substantially elevated in those with type 1 and type 2 diabetes compared with the background population
8	Holman et al.	2020	United Kingdom (England)	Original Article	To ascertain the associations between risk factors (hyperglycaemia and obesity etc.) and COVID-19-related mortality in people with type 1 and type 2 diabetes was assessed	Deaths in people with type 1 and type 2 diabetes rose sharply during the initial COVID-19 pandemic in England. Increased COVID-19-related mortality was associated independently with glycaemic control and BMI
9	Hamer et al.	2020	United Kingdom	Original Article	To examine the prospective association of diabetes and glycaemic control with COVID-19 hospitalisation in a large community-based cohort study	It was established that higher levels of A1C within the normal range were a risk factor for COVID-19
10	Akbariqomi et al.	2020	Iran	Original Article	To describe epidemiological and clinical characteristics along with outcomes of hospitalized COVID-19 patients with and without diabetes	COVID-19 patients with diabetes had more co-morbidities, are at a higher risk of complications and had a higher in-hospital mortality than non-diabetic patients
11	Bramante et al.	2020	America	Original Article	To identify whether metformin reduced COVID-19-related mortality and whether sex-specific interactions exist	Metformin was significantly associated with reduced mortality in women with obesity or type 2 diabetes who were admitted to hospital for COVID-19
12	Lassale et al.	2020	United Kingdom	Original Article	To examine the role of socioeconomic, mental health, and pro-inflammatory factors in a community-based sample. This is because the differentials in COVID-19 hospitalisations and mortality according to ethnicity has uncertain origins	There were clear ethnic differences in the risk of COVID-19 hospitalisation but, these were not fully explained by measured factors
14	Sheshah et al.	2020	Saudi Arabia	Original Article	To characterize comorbidities and associated risk factors with mortality among hospitalized adults with COVID-19	Type 2 diabetes mellitus (T2DM) was the most common comorbidity in COVID-19 patients. The male gender and South Asian ethnicity were significant comorbidities and dexamethasone improved outcomes
13	Goldman et al.	2020	United Kingdom	Original Article	To describe the prevalence of diabetic ketoacidosis in individuals admitted to a single centre with COVID-19	It was concluded that diabetic ketoacidosis is common and severe in individuals hospitalised with COVID-19
14	Chan et al.	2020	America	Original Article	To explore the clinical characteristics and outcomes of COVID-19 patients presenting with combined diabetic ketoacidosis and hyperosmolar hyperglycaemic state	The data showed that diabetic patients are at risk of developing combined diabetic ketoacidosis & hyperosmolar hyperglycaemic state, which is associated with COVID-19 and a substantial mortality
15	Wang et al.	2020	China	Original Article	To describe the epidemiological and clinical characteristics of NCIP	Within this study the presumed hospital-related transmission of 2019-nCoV was suspected in 41% of patients, 26% of patients received ICU care, and mortality was 4.3%
16	Guan et al.	2020	China	Original Article	To evaluate the risk of serious adverse outcomes in patients with COVID-19 by stratifying the comorbidity status	There is a correlation between a greater number of comorbidities with poor clinical outcomes. Patients with any comorbidity yielded poorer clinical outcomes than those without
17	Martin et al.	2020	England	Original Article	To investigate whether minority ethnicity and occupational factors influence anti-SARS-CoV-2 IgG seroprevalence in hospital staff	Ethnicity and occupational factors, including specialty and seniority, are associated with seropositivity for anti-SARS-Cov-2 IgG. These findings could be used to inform occupational risk assessments for front-line healthcare workers
18	Harrison et al.	2020	USA	Original Article	To determine associations between comorbidities listed in the Charlson comorbidity index and mortality among patients in the United States with COVID-19	Identifying patient characteristics and conditions associated with mortality with COVID-19 is important for hypothesis generating for clinical trials and to develop targeted intervention strategies
19	Shah et al.	2020	USA	Original Article	To describe the demographics, and outcomes of hospitalized COVID-19 patients in rural Southwest Georgia	Patients hospitalized with COVID-19 in rural US have higher comorbidity burden. Immunosuppression, hypertension, age ≥ 65 years, and morbid obesity are independent predictors of increased mortality. Female gender is an independent predictor of reduced mortality
20	Best et al.	2020	USA	Original Article	To describe baseline demographics and clinical characteristics of US patients hospitalized with COVID-19 and pulmonary involvement	Compared with white patients, African American patients were younger, with higher BMI, higher prevalence of concurrent diabetes, and lower prevalence of COPD and smoking/tobacco use
21	Prado-Galbarro et al.	2020	Mexico	Original Article	To evaluate the association of chronic diseases and indigenous ethnicity on the poor prognosis of outpatients with coronavirus disease 2019 (COVID-19) and hospitalised patients in Mexico	Diabetes, hypertension, and obesity combined with older age, male sex and indigenous ethnicity increase the risk of death after SARS-CoV-2 infection in the Mexican population
22	Castelnuovo et al.	2020	Italy	Original Article	To investigate factors that predispose patients to a higher in-hospital death risk at 30 Italian clinical centres, using data from the CORIST Collaboration	Factors that pre-disposed patients to a higher risk of in-hospital death (in Italy) included impaired renal function, elevated levels of CRP and advanced age
23	Palaiodimos et al.	2020	USA (New York)	Original Article	To investigate if obesity is associated with worse in-hospital outcomes for patients To assess and present the clinical characteristics and early outcomes of patients who were diagnosed with COVID-19 and admitted to a large tertiary academic centre	In hospital mortality was 24%, and after a 21-dy follow up only 3% of patients were still hospitalised Factors that were independently associated with mortality requiring intubation included severe obesity, increasing age, and male sex Factors that were independently associated with increasing oxygen requirements during hospitalisation included severe obesity, increasing age, male sex and smoking
24	Lusignan et al.	2020	United Kingdom	Original Article	To identify demographic and clinical risk factors for testing positive for SARS-COV-2 within a primary care surveillance programme	Risk factors that increased the odds of a positive SARS-COV-2 test included increasing age, male sex, increasing deprivation, urban location, and black ethnicity Clinical risk factors that were independently associated with a positive SARS-COV-2 test included chronic kidney disease and increased BMI
25	Bhatti et al.	2020	Dubai, UAE	Original Article	To describe the clinical characteristics and outcomes of patients with diabetes that were admitted to a Dubai hospital for treatment of moderate-to-severe COVID-19	Patients that have diabetes that are male, part of the BAME population, and that other comorbidities such as cardiovascular disease are at a higher risk of developing severe outcomes associated with COVID-19
26	Richardson et al.	2020	USA	Original Article	To describe the clinical characteristics and outcomes of patients with COVID-19 hospitalized in the US health care system	In the study patients that were hospitalised from COVID-19 were mostly men, older people, or those with diabetes or hypertension. Out of the patients that were younger than 20 years, none of them died. The mortality rate was lower in females compared to males for every 10-year age interval above 20 years
27	Zhou et al.	2020	China	Original Article	To explore risk factors of in-hospital mortality and describe the clinical course of symptoms, viral shedding, and temporal changes of laboratory findings during hospitalisation	Increased age was associated with death in COVID-19 patients. Higher SOFA score, increased d-dimer levels at admission and older age were the risk factors for mortality in adult COVID-19 patients
28	Petrilli et al.	2020	USA	Original Article	To describe outcomes of people admitted to hospital with COVID-19 in the USA, and the clinical and laboratory characteristics associated with severity of illness	The majority of people admitted to hospital were men and were more likely to have comorbidities such as diabetes, cardiovascular disease, and chronic kidney disease. Age and comorbidities are predictors of requirement for hospital admission instead of outpatient admission. Markers of inflammation and oxygen impairment has been linked to poor outcomes in hospital admission
29	Chen et al.	2020	China (Wuhan)	Original Article	To describe the outcomes and clinical characteristics of patients with diabetes in whom COVID-19 has been confirmed or clinically diagnosed, and their association with glucose-lowering or blood pressure–lowering medications	Elevated C-reactive protein and old age were risk factors for higher mortality in COVID-19 patients with diabetes. Usage of insulin has been associated with poor prognosis
30	Alguwaihes et al.	2020	Saudi Arabia	Original Article	To find more information on the clinical characteristics and outcomes of hospitalized COVID-19 patients with or without diabetes mellitus in Saudi Arabia	Hospitalised COVID-19 patients in Riyadh had a high prevalence of diabetes mellitus. Diabetic patients had increased mortality. Old age, smoking, congestive heart failure, use of β-blockers, elevated creatinine and severe vitamin D deficiency have been linked to fatality in these patients
31	Fox et al.	2020	USA	Original Article	To investigate the relationship between diabetes and COVID-19 mainly in the African American population	Patients with diabetes mellitus and COVID-19 had poorer outcomes and more severe disease. Age was associated with mortality. There was no difference in outcomes and severity of disease when comparing African American patients with non-African American patients
32	Ioannou et al.	2020	USA	Original Article	The study aimed to find the risks associated with hospitalization, mortality and mechanical ventilation whilst having SARS-CoV-2	In the cohort of veterans that tested positive for SARS-CoV-2, hospitalisation, mortality, and mechanical ventilation was higher
33	Gu et al.	2020	USA	Original Article	What are the sociodemographic risk factors of COVID-19 and do they differ by ethnicity or race	There is a racial disparity with COVID-19 that cannot be explained even with controlling sex, age, comorbidity score, socioeconomic status to which target interventions to support higher risk populations are needed
34	Ebinger et al.	2020	USA	Original Article	The study aimed to find the clinical characteristics and the demographic associated with the increased severity of COVID-19	In the UK health care system, there is a greater severity of COVID-19 in patients who are African, older, male, obese, American, with diabetes and with overall greater comorbidity burden
35	Clark et al.	2020	United Kingdom	Original Article	The risk of getting COVID-19 is higher in people who are older and who have underlying health conditions so understanding the figures between each country should help give provide a strategy to help shield or vaccinate those	People with underlying health condition have a 1 in 5 chance of developing severe COVID-19 should they get it
36	Atkins et al.	2020	United Kingdom	Original Article	The study looks at whether being older, having hypertension, coronary heart disease, diabetes are true risk factors of getting COVID-19	People who are older have higher risk comorbidities to increase the risk of getting COVID-19 rather than it being simply age-related
37	Haywood et al.	2020	USA (Louisiana)	Original Article	To compare the clinical characteristics and hospital course of laboratory-confirmed cases of COVID-19 amongst black non-Hispanic and white non-Hispanic subpopulations in Louisiana	Blacks represented the majority of all COVID-19 positive patients, and had higher prevalence of obesity, diabetes, hypertension, and chronic kidney disease than white patients A larger percentage of black patients has elevated levels of creatinine, AST or inflammatory markers than white patients Increased odds of hospital admission were associated with black race, increasing age, a higher score on the Charlson comorbidity Index, public insurance, residence in a low-income area, and obesity The risk of in-hospital death is associated with age, respiratory rate, and several biomarkers (levels of venous lactate, creatinine, procalcitonin and platelet count)
38	Soares et al.	2020	Brazil	Original Article	To analyse the relationship of clinical factors, comorbidities and demographic characteristics against hospitalisation and death from COVID-19	Factors that increased the risk of hospitalisation include older age, male gender, Asian, indigenous, or unknown race, all comorbidities (smoking, kidney disease, obesity, pulmonary disease, diabetes, and cardiovascular disease), fever and shortness of breath Factors that increased death outcomes for hospitalised patients included older age and shortness of breath
39	Yan et al.	2020	China (Wuhan)	Original Article	To investigate the clinical characteristics of patients with severe COVID-19 with diabetes mellitus, and the association of diabetes with the outcomes in patients that have severe COVID-19	Patients with severe COVID-19 and diabetes are more likely to receive admission to the ICU and have a higher mortality Severe inflammatory response was seen in patients with both COVID-19 and diabetes Diabetes is a risk factor for death for patients with severe COVID-19
40	Sardu et al.	2020	Italy	Original Article	To investigate whether poor glycaemic control was associated with poor outcomes and whether early optimal glycaemic control along with hospitalisation reduces plasma IL-6 and D-dimer levels, therefore improving the outcomes for hospitalised patients with COVID-19	Optimal glycaemic control during hospitalisation has been associated with a reduced risk of severe disease and death in patients with COVID-19 Patients who were hyperglycaemic showed a higher incidence of severe disease and higher levels of IL-6 compared to patients that were normoglycemic Patients with hyperglycaemia who developed severe disease presented higher D-dimer levels compared to those with normoglycemia

## Results

The total number of articles retrieved from each database using the relevant keywords included 463 articles (Pubmed = 55, Google Scholar = 75, Science direct = 198, Scopus = 3, Springer Link = 132). Duplicates and irrelevant articles were removed according to the inclusion and exclusion criteria, resulting in 156 articles. The 156 articles were screened in compliance with the PRISMA checklist, which identified a total number of 40 relevant articles that were included in the study. In an analysis of 40 papers, two main themes were identified. These are: (a) COVID-19 in diabetic patients is associated with high mortality, and (b) Patients’ characteristics could influence COVID 19 symptoms in diabetic patients. 

### COVID-19 in diabetic patients is associated with high mortality

Research by Holman and his colleagues showed that mortality rate of diabetic patients with COVID-19 is higher than non-diabetic patients [2]. According to the Office for National Statistics from January 2, 2017, to May 11, 2020, in England, out of 1604 people with type 1 diabetes who died from all causes, 464 of these deaths were related to COVID-19 [2]. Furthermore, 36,291 people with type 2 diabetes died from all causes, and 10,525 of these deaths were COVID-19 related; the percentage of deaths in diabetic patients is approximately 29% [2]. Compared to previous years, this study showed that the number of people who died of diabetes was significantly higher due to the emergence of COVID-19 [2].

Studies in Scotland indicated that of 319,349 diabetic people, 2724 people contracted COVID-19 and 1082 had severe symptoms. However, of the non-diabetic people, 4081 people (0.1%) from 5,143,951 suffered from severe or fatal COVID-19 [3].

This is supported by a study conducted in a hospital in Iran, where among the 595 COVID-19 patients, 148 patients had diabetes. Patients with diabetes were found to have more comorbidities and complications compared to those without diabetes, which required them to have more respiratory support [4]. The rise in these complications could possibly be due to the detrimental effects of the SARS-CoV-2 virus on the immune system, as diabetic patients already have increased levels of inflammatory markers, which could explain why diabetic patients have higher mortality rates if they contracted Covid 19. This shows that the mortality rate in COVID-19 patients with diabetes is still considerable and the inflammatory response in diabetic patients is much more severe compared to the background population [5–7].

Another study reported that 6256 patients were hospitalized with COVID-19 and many patients were patients with type 2 diabetes. These patients were divided into two groups, one group was treated with metformin while the other group was in the non-metformin group. Of the 2333 patients who were in the metformin group, 394 of them died, indicating a mortality rate of 16.9%. 3923 patients were in the non-metformin group and 791 of them died, indicating a mortality rate of 20.2%. As a result, metformin was linked to a decrease in mortality in COVID-19 patients in diabetic patients [8].

### Patients’ characteristics could influence covid 19 symptoms in diabetic patients

The studies showed variations in symptoms in relation to the characteristics of the patients. In this theme, four subthemes were identified: older diabetic patients suffer from severe symptoms, males are more likely to be badly affected, non-white diabetic patients are at higher risk, the higher the BMI, the higher the risk in diabetic patients.

The results also showed that age contributes to the severity of COVID-19 symptoms. The results showed that older age and being male increased the chance of fatality from COVID-19 for type 1 and type 2 diabetic patients [2]. In another study, 2.8% of the people who developed severe COVID-19 were under 50 years old, while 97.3% were over 50 years old. The results of the study also showed that men were more likely to have severe COVID-19 than women (0.4% vs 0.3%). Furthermore, 51 of 34,383 people (0.1%) that had type 1 diabetes and 1008 people (0.4%) who had type 2 diabetes suffered from severe or fatal COVID-19 [3].

Studies have also shown that different underlying health complications and comorbidities can further exacerbate the state of a patient’s disease. A study carried out in hospitalized COVID-19 patients in Mexico showed that of 15,529 patients, 62.6% were over the age of 40, 57.8% were men and a high proportion were diagnosed with diabetes (18.4%), hypertension (21.9%) and obesity (20.9%) [9]. This finding is similar to other studies that have focused on COVID-19 risk factors where it was found that the key characteristics of COVID-19-dead patients included older age, male, as well as comorbidities such as diabetes, obesity, hypertension, cardiovascular disease, and respiratory disease [10–13]. This implies that older age and male in conjunction with underlying health comorbidities such as diabetes, hypertension, and obesity can increase the risk of mortality in COVID-19 patients and correlate with poor clinical outcomes, as they can make an individual more susceptible to an inflammatory reaction that results in rapid progression of the disease and adverse prognosis of COVID-19. This statement is also supported by other sources [14–27], which emphasizes the importance of maintaining a healthy lifestyle during the pandemic to reduce the risk of developing underlying comorbidities [28].

Patients who had diabetes were older, more prone to receiving mechanical ventilation and ICU, and had higher mortality. The clinical characteristics of these patients included elevated D-dimer levels, increased white blood cell count, neutrophil count, C-reactive protein as well as having a more severe inflammatory response [29].

In Iran, 595 patients with COVID-19 with diabetes were hospitalized, of these patients were men and the median age was 55 years, 148 of the patients (24.9%) were diabetic and those who had diabetes also had more comorbidities than non-diabetic patients such as hypertension (48.6% vs 22.3%), chronic liver disease (9.4% vs 4%), chronic kidney disease (16.2% vs 7.6%) and cardiovascular disease (27% vs 16.1%) [4]. Laboratory test results showed that the diabetic patients had higher white blood cell counts compared to non-diabetic patients but had lower red blood cell counts. While the patients were in hospital, 511 patients (86%) received oxygen therapy; in a form of inhalation, non-invasive ventilation, or invasive mechanical ventilation 402, 81 and 28, respectively. Complications included acute respiratory distress syndrome (ARDS), shock, and secondary infections and from the patients—65 died, 156 were discharged, and 374 remained in hospital. When comparing the complications, the study showed that patients with diabetes were more likely to have suffered ARDS (19.2% vs 8.7%), shock (17.6% vs 6.5%), and secondary infection (19.2% vs 8%) than non-diabetic patients [4]. It has been observed that diabetic patients needed more oxygen compared to non-diabetic patients (80.4% vs 63.3%) and the figures showed the same pattern for the different ventilations.

Ethnicity was seen to have a potential link in the worsening prognosis in COVID-19. The anti-SARS-CoV-2 IgG seroprevalence of South Asian and Black hospital staff was reported to be significantly higher than that in white staff [30]. Some studies reported that individuals from non-White ethnic groups were associated with an increased risk of COVID-19 infection, hospitalisation, and ICU admission) compared to the White ethnic group [31, 32]. Black patients were more likely to be hospitalized, need intubation, received mechanical ventilation, and had an increased mortality rate when compared with White patients [11, 33, 34]. It was also reported that the Black and Hispanic ethnicities were significantly associated with testing positive for COVID-19. These results suggested that individuals from minority ethnic groups are associated with increased risk of infection with COVID-19 and ethnic minority individuals have worse/negative clinical/disease outcomes when compared to the majority ethnic group [35]. On the other hand, studies have reported that in hospitalized patients with COVID-19, black and Hispanic ethnicities were not significantly associated with mortality [27, 33, 34]. However, although these results suggest that black and Hispanic ethnicities are not associated with increased mortality in COVID-19 patients, they do not diminish the risk of negative outcomes in black and Hispanic individuals other than death.

Further studies reported that Black individuals had more COVID-19 risk factors than white individuals and that hospitalised Black COVID-19 patients had more chronic diseases when compared to other ethnicities [35, 36]. COVID-19 comorbidity risk factors included obesity/higher BMI, diabetes, hypertension, and chronic kidney disease, which were more prevalent in Black patients compared to White patients [33, 35, 37]. Adiposity/obesity was more strongly associated with an increased risk of positive tests and death in non-white COVID-19 patients compared to white individuals [38].

A study carried out on 872 patients with 48.1% Black, 33.7% White, 5.6% Asian and 12.6% mixed/other Asian reported a higher risk ratio of 3.12 in the Black ethnic group compared to the White population which had a hazard ratio of 2.97 with black people between the ages of 45 and 65 having an especially high rate of hospital admissions [39]. Moreover, the prevalence of hypertension, diabetes, chronic kidney disease, and obesity were higher amongst Black patients compared to White patients with COVID-19. Diabetes and obesity were also more common among Asian patients compared to patients who were White [39].

At the University Hospitals of Leicester (UHL) NHS Trust, Martin et al. reported that with conditions such as diabetes, 11.6% and 12.7% of Black males and females had the disease, respectively. This percentage was lower in the white male and female groups with 10% and 8.5%, respectively [30]. Higher mortality was reported in Blacks and Asians than in White ethnic groups diagnosed with T1DM; the reported hazard ratio for Black was 1.77, for Asians 1.57 and 1 for Whites [2]. Furthermore, differences in the number of hospital ICU admissions were reported in different ethnic groups, the hazard ratios for Indians, Blacks and White were 2.37, 2.89, and 1, respectively [32, 33]. A study in Dubai also showed a link between males and people with the BAME having a worse prognosis with COVID-19 than those who are not [20].

Other studies further confirmed that patients of Black and Asian background with type 1 diabetes had an increased risk of mortality compared to White people [2]. For type 2 diabetes, the deaths from COVID-19 were a lot higher in people from BAME ethnicity. The results also showed that patients who had hyperglycaemia before had a strong link with COVID-19 related death. This was shown as type 2 diabetic patients who had a HbA1c of 59 mmol/mol had a higher chance of COVID-19 mortality than those with lower levels. In this study, patients with type 1 and type 2 diabetes had an increased chance of mortality if they were obese, especially people of Asian and Black ethnicities. Having impaired renal function also increased the number of deaths in both types of diabetic patients. Additionally, hypertension (systolic pressure over 140 mm HG) was linked to COVID-19 mortality in people with type 2 diabetes in Asian and Black ethnicities. Other comorbidities that were reported such as stroke and heart failure, which further increased the risk of COVID-19 mortality in type 1 and type 2 diabetic patients. The study results also showed that type 2 diabetes patients taking at least one hypertensive drug had an increased chance of COVID-19 mortality, while statins reduced mortality rates [2].

HbA1c and CRP levels, as well as the incidence of diabetes, were higher in Black and Asian individuals than in White individuals, even at a younger age. Patients of Black ethnicity were 4 times as likely to be hospitalized for COVID-19, and Asian and other ethnic groups were twice as likely to be hospitalised for COVID-19, compared to patients who were White [32]. According to other studies, Black patients were also observed to be more likely to test positive for COVID-19 (41.8% vs 13.2%) and were more likely to be admitted to the hospital with COVID-19 (52.7% vs 38.6%) than White patients. In White patients, more comorbidities were associated with hospitalisation for COVID-19, whereas this was not the case for Black patients [34].

## Discussion

Attempts were made to review the literature focusing on diabetic patients who experienced complications when they contracted COVID-19, as well as the impact of ethnicity, which may play an important role in terms of the development of symptoms and their severity and the response to medications. Although it was found that diabetes is a risk factor for COVID-19, many people with diabetes also have other underlying health conditions, therefore this should be considered as a possible contributing factor for the worse outcomes of covid-19 in people with diabetes. It was found that at least 40% of patients with diabetes had at least 1 microvascular comorbidity (renal disease, neuropathy, diabetic eye disease), 79% at least 1 macrovascular comorbidity (coronary disease, cerebrovascular disease, hypertension, heart failure), and 61% at least 1 non-diabetes-related comorbidity (lung disease, cancer, arthritis) [32]. In addition to this, it was found that patients with multiple comorbidities had poorer management of their diabetes, which with other comorbidities further increased their risk of poorer COVID-19 outcomes. Patients with diabetes have a more severe inflammatory response, leading to poorer outcomes associated with COVID-19 compared to patients without diabetes [32]. Although in another study, it was reported that patients with diabetes, but without other comorbidities, were still at increased risk of severe pneumonia, release of enzymes related to tissue injury, excessive uncontrolled inflammation responses, and hypercoagulable state associated with dysregulation of glucose metabolism. In these patients, serum levels of inflammatory biomarkers such as IL-6, C-reactive protein (CRP), ferritin and D-dimer were significantly higher [40]. This suggested that patients with diabetes are more likely to experience the ‘cytokine storm’ and a more severe inflammatory response, which ultimately leads to worse outcomes associated with COVID-19. Therefore, it can be suggested that diabetes alone is a risk factor for COVID-19 and that the complications that arise are not due to other comorbidities. In addition, the levels of neutrophils were abnormally high in these patients, and the levels of lymphocytes were lower, again suggesting a higher risk of inflammatory responses and hypercoagulable state leading to worse outcomes [4, 40].

Black and Asian individuals were at increased risk for diabetes and were a risk factor for worse outcomes associated with COVID-19. This suggested that Black and Asian people are likely to be predisposed to worse outcomes and an increased risk of hospitalization with COVID-19 [13]. People with diabetes are likely to have more than one comorbidity, and people from ethnic minorities are more likely than White people to have diabetes. This suggests a correlation between ethnicity and the risk of developing diabetes, which in turn increases the chance of developing other comorbidities. Previous studies have suggested that people of ethnic minorities are more likely to have comorbid conditions than white people. As people of ethnic minorities are more likely to suffer from multiple comorbidities, this also suggests that they are at higher risk of developing COVID-19. Although studies found that more comorbidities were associated with COVID-19 in White patients but not in Black patients, the higher prevalence of comorbidities in Black patients may still put them at increased risk of poorer outcomes associated with the virus [33].

Studies also showed that there was a significantly higher number of patients with type 2 diabetes (10,525 patients) that died from COVID-19 compared to patients with type 1 diabetes (464 patients) [2]. Since the incidence of diabetes was higher in Black and Asian individuals even at a younger age compared to White patients, this suggests that type 2 diabetes more than type 1 diabetes pre-disposes people of a Black or Asian ethnic background to worse outcomes and a higher risk of mortality associated with COVID-19 [41].

Interestingly, male gender was a risk factor for COVID-19 mortality and increased oxygen requirements [42]. Males are at a much higher risk of poor outcomes associated with COVID-19 compared to women. Evidence has shown that men are at a higher risk of developing diabetes at a younger age and a lower BMI compared to women [43]. Therefore, a higher prevalence of diabetes among men may play a significant role in predisposing the male sex to increased COVID-19 mortality.

In addition, studies have shown that patients that have chronic kidney disease (CKD) are more likely to test positive for COVID-19 and are at an increased risk of mortality from COVID-19 [19, 42]. The kidneys contain ACE-2 Receptors, which allows the virus to directly target the kidneys and cause damage to the podocytes. Many patients who develop COVID-19 also develop acute Kidney Injury, and studies have shown that patients who have a history of CKD are at an increased risk of developing acute kidney injury [44]. In fact, 35% of patients who developed acute kidney injury had a history of CKD [46]. Furthermore, studies have shown that patients with COVID-19 have shown reduced kidney function, characterised by abnormal levels of proteins in the urine (proteinuria) and the presence of blood in the urine (haematuria) [44]. Additionally, diabetes is the most common cause of chronic kidney disease.

Interestingly, studies have shown the transmembrane protease serine 2 (TMPRSS2) to be expressed at a higher level within the nasal epithelium in Black people compared to other ethnicities. The SARS-CoV-2 virus uses TMPRSS2 as a host for S protein priming and fusion of viral and host cell membranes, which could explain why there is a higher infection burden amongst Black people [46].

Other published data have also shown that patients with diabetes and hyperglycaemia have a greater risk of being more severely affected by SARS-CoV-2 compared to those with normoglycemia and who are non-diabetic [48]. A study investigating the impact of glycaemic control on the severity of COVID-19 found that patients with an HbA1c of 86 mmol/mol or greater had a higher risk of COVID-19-related mortality than those with a lower HbA1c (47–52 mmol/mol) [2]. This shows that diabetic patients are more at risk of developing a hyperglycaemic state which could have an impact on the rate of COVID-19 related mortality and the severity of infection. This finding is similar to other studies that have focused on risk factors for COVID-19 [3, 47–50]. It was also shown that patients who had hyperglycaemia also had elevated IL-6 and D-dimer levels [48]. This implies that glycaemic control is a significant factor in the severity of SARS-CoV-2 infection, and COVID-19 patients who have elevated HbA1c levels tend to have a more severe inflammatory response.

## Limitations

Although this study contains credible information highlighting the impact of COVID-19 in conjunction with diabetes and relevant risk factors and treatment strategies, there were several limitations in this review. First, this study only included original research articles and excluded secondary studies, thus preventing a variety of expert perspectives and insights. The studies included within this review were from different countries, which could insinuate a lack of under reporting and thus impact the quality of data. The predefined inclusion criteria meant that articles published between January 2019 to December 2020 could only be included, which could have meant that we missed out on additional insights and key findings on COVID-19. Furthermore, due to the rapidly changing management of COVID-19, some of the findings and results could be affected. However, despite these limitations, this review does provide key information and insights on the impacts of COVID-19 in correlation with diabetes, and the associated risk factors and therapeutic approaches used to manage and/or prevent progression of the disease and the adverse effects of SARS-CoV-2.

## Conclusions

The accumulated data confirmed that patients with diabetes are at an increased risk of COVID-19, some of the risk factors for these patients included BAME ethnicity, high BMI, male gender and older age. Patients with these risk factors were predisposed to worse outcomes associated with COVID-19. More research is required to investigate additional risk factors for people with diabetes that may predispose them to worse outcomes associated with COVID-19. Although attempts to vaccinate people have progressed rapidly, further research is needed to consider the speed with which the virus is mutating and the fact that some individuals may not respond well to vaccination.
